# Piecewise-Constant-Model-Based Interior Tomography Applied to Dentin Tubules

**DOI:** 10.1155/2013/892451

**Published:** 2013-02-21

**Authors:** Peng He, Biao Wei, Steve Wang, Stuart R. Stock, Hengyong Yu, Ge Wang

**Affiliations:** ^1^The Key Laboratory of Optoelectronic Technology and Systems of the Education Ministry of China, Chongqing University, Chongqing 400044, China; ^2^Biomedical Imaging Division, VT-WFU School of Biomedical Engineering and Sciences, Virginia Tech, Blacksburg, VA 24061, USA; ^3^Argonne National Laboratory, 9700 South Cass Avenue, Argonne, IL 60439, USA; ^4^Department of Molecular Pharmacology and Biological Chemistry, Feinberg School of Medicine, Northwestern University, 303 East Chicago Avenue, Chicago, IL 60611-3008, USA; ^5^Biomedical Imaging Division, VT-WFU School of Biomedical Engineering and Sciences, Wake Forest University Health Sciences, Winston-Salem, NC 27157, USA

## Abstract

Dentin is a hierarchically structured biomineralized composite material, and dentin's tubules are difficult to study in situ. Nano-CT provides the requisite resolution, but the field of view typically contains only a few tubules. Using a plate-like specimen allows reconstruction of a volume containing specific tubules from a number of truncated projections typically collected over an angular range of about 140°, which is practically accessible. Classical computed tomography (CT) theory cannot exactly reconstruct an object only from truncated projections, needless to say a limited angular range. Recently, interior tomography was developed to reconstruct a region-of-interest (ROI) from truncated data in a theoretically exact fashion via the total variation (TV) minimization under the condition that the ROI is piecewise constant. In this paper, we employ a TV minimization interior tomography algorithm to reconstruct interior microstructures in dentin from truncated projections over a limited angular range. Compared to the filtered backprojection (FBP) reconstruction, our reconstruction method reduces noise and suppresses artifacts. Volume rendering confirms the merits of our method in terms of preserving the interior microstructure of the dentin specimen.

## 1. Introduction

Teeth are important and interesting biomineralized tissues with remarkable mechanical properties through their hierarchy of structures [1]. Enamel, a hard, resistant material almost totally composed of carbonated apatite (cAp), covers the outer, exposed portion of the tooth. The tooth's interior (and the majority of its volume) consists of dentin, a tough composite of carbonated apatite (cAp) and collagen. Prominent features within dentin are the tubules that extend from near the junction with enamel to the pulp cavity in the tooth's interior. Tubule diameters are typically 1-2 *μ*m, and tubule spacing is ~5–10 *μ*m. Smaller channels called canaliculi run from the tubules into the surrounding dentin, and their diameters are in the range of 100–300 nm [2].

Dentin tubules and their surroundings remain of interest not just to microanatomists but also to those studying the efficacy of prostheses' attachment. The small dimensions of dentin tubules make them difficult to evaluate and have motivated major research efforts. Up to date, tubules and their surroundings have been characterized with microradiography [3], scanning electron microscopy (SEM) [4], transmission electron microscopy (TEM) [5], secondary ion mass spectroscopy (SIMS) [6] position-resolved X-ray diffractometry [7], micro-CT [8, 9] and nano-CT [10, 11]. With the exception of the CT techniques, these techniques provide essentially 2D views of intrinsically 3D tubules. 

Of particular interest, nano-CT provides the requisite 3D spatial resolution for studying dentin's tubules and canaliculi. However, exact nano-CT reconstruction typically requires that the specimen remains within the field-of-view (FOV) during a 180° scan. Suitable cross sections, say 25 *μ*m across, can be machined from thin wafers of dentin using focused ion beams, but this process is quite slow and would limit the number of tubules that could be examined. Local or region-of-interest (ROI) tomography is quite valuable for reconstructing samples larger than the FOV but requires reasonable X-ray transmissivity for all projection directions and would be subject to the throughput limitations. Plate-like samples 25–50 *μ*m thick and millimeters in lateral extent would contain hundreds of tubules extending significant lengths, but any attempts at nano-CT reconstruction would suffer from significant data truncation and angular limitation, significantly degrading image quality. If plate-like dentin samples could be used for nano-CT, dozens of tubules would be imaged without extraordinary sample preparation efforts. Such datasets would greatly improve understanding of tubule morphology and its variability within a tooth. Accordingly, we performed synchrotron nano-CT on a thin dentin plate and reconstructed its microstructure using an interior tomography method.

In classic CT theory, an interior ROI cannot be reconstructed exactly from truncated projections. As a result, features outside the ROI may seriously disturb the features inside the ROI, often hiding or distorting vital information. A recent progress demonstrated that the interior problem can be exactly and stably solved if a subregion in the ROI is known [12–15], which is referred to as interior tomography. However, it can be difficult to obtain precise prior knowledge of a sub-region in many cases. A further progress in interior tomography was inspired by compressive sensing (CS) theory. The main idea of CS theory is that an image can be reconstructed from a rather limited amount of data as long as an underlying image can be sparsely represented in an appropriate domain and determined from these data [16–18]. Inspired by CS theory, it was found that an interior ROI can be exactly and stably reconstructed via the total variation (TV) minimization if the ROI is piecewise constant [19, 20]. 

For dentin image reconstruction, we employed an ordered-subset simultaneous algebraic reconstruction technique (OS-SART) to reconstruct the dentin slice images [21–24]. After analyzing the characteristics of the dentin slices, we found that the dentin slice can be divided into two types of regions: the pores and the dentin between the pores. The attenuation coefficient inside the pores differs from that of the dentin, but both are approximately constant. Therefore, the sample images can be sparsified by a discrete gradient transform (DGT), and the total variation (TV) minimization can be used to reconstruct high-quality dentin images from truncated projections even if the angular range of a scan is limited, as we observed in this project. 

This paper is organized as follows.  Section 2 describes a specimen and data acquisition, characteristics of the data, and our interior tomography algorithm.  Section 3 compares the reconstructions by interior tomography and filtered backprojection (FBP) methods.  Section 4 discusses relevant issues and concludes the paper.

## 2. Materials and Methods

### 2.1. Sample Preparation

A thin wafer of bovine dentin was cut from a molar using a diamond wafering saw (Isomet 1000, Buehler, Lake Bluff, IL) to a thickness of about 150 *μ*m. The wafer was thinned to ~25–50 *μ*m by polishing with 1000 grit SiC paper between two glass microscope slides. Its lateral dimensions were greater than 2 mm.

### 2.2. Nano-CT

Compared to medical and micro-CT, nano-CT uses an X-ray lens to bring spatial resolution into the nanometer domain. To date, better than 20 nm has been achieved for routine use with multi-keV hard X-ray radiation which is able to penetrate hundreds of microns of dental tissue [25].

**Figure 1:**
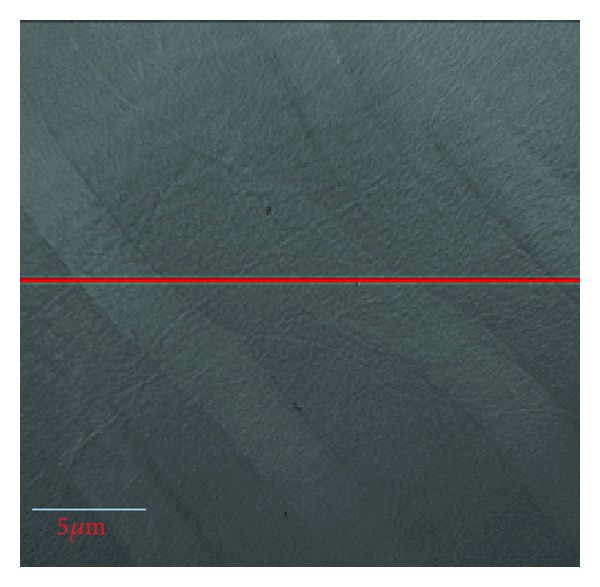
One truncated projection of the dentin specimen.

**Figure 2:**
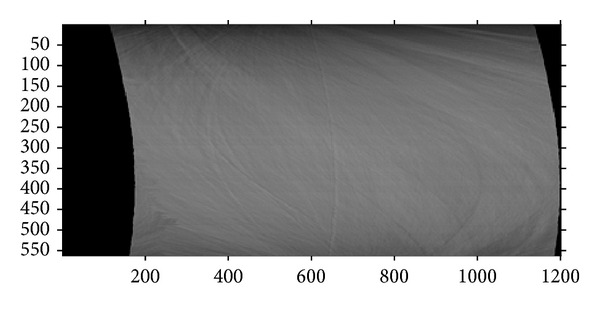
Sinogram consisting of 561 truncated projections for the slice marked by the red line in Figure 1.

The dentin specimen was imaged by the transmission X-ray microscope at Sector 32-ID of the Advanced Photon Source, Argonne National Laboratory, USA. The synchrotron nano-CT system can be viewed as in a typical parallel-beam geometry and employs monochromatic 8 keV X-radiation. The X-ray detector contained 2048 × 2048 pixels with 12.5 nm pixel size and 25 × 25 micron FOV. The angular scanning range for nano-CT was ±70° with a 0.25° steps, producing 561 projections. The voxel size in the reconstruction was 12.5 nm. Due to the small FOV of the nano-CT system, the X-ray beam could not cover the specimen completely, and all of the projections were truncated.  Figure 1 shows a truncated projection of the specimen in which several tubules can be clearly seen running from upper left to lower right.  Figure 2 is an extracted sinogram for one image slice along the marked line in Figure 1. Preliminary reconstructions revealed that the specimen consisted of two types of material, pores (tubules and canaliculi) and the dentin between the pores. The specimen is, therefore, approximately piecewise constant.

### 2.3. Reconstruction Algorithm

The conventional CT approach cannot exactly reconstruct an internal ROI only from truncated projections through the ROI because this interior problem does not have a unique solution in an unconstrained setting. Interestingly, recent results show that the interior problem is solvable if appropriate yet practical prior information is available. In particular, if the attenuation coefficient distribution on a small sub-region in an ROI is known, or the attenuation coefficient distribution over the ROI is piecewise constant, the interior problem has a unique solution. Theoretically, a function can be well approximated by piecewise constant functions, so the present dentin specimen is modeled as being piecewise constant. In this project, the piecewise-constant-model-based interior tomography algorithm was used to reconstruct dentin images from truncated projections over a limited angular range. The interior tomography algorithm is robust against noise by minimizing the image TV. Specifically, we employed the ordered-subset simultaneous algebraic reconstruction technique (OS-SART) for interior reconstruction of the dentin specimen.

The imaging process can be modeled as a linear system in terms of the popular pixel basis functions: *Af* = *b*, where  *b* = (*b* ^1^,…, *b* ^*M*^) ∈ *R* ^*M*^ represents the truncated projection data with *M*  being the total projection number, *f* = (*f*_1_,…, *f*_*N*_) ∈ *R* ^*N*^  denotes an object to be reconstructed with *N*  being the total pixel number, and *A* = (*a*_*ij*_) is the system measurement matrix with  *i* = 1,…, *M*,  *j* = 1,…, *N*. The major algorithmic steps are described as follows.

While the ART method is the first iterative algorithm used for CT reconstruction [26], the SART is a major refinement to the ART [27]. In recent years, some advanced techniques were developed to accelerate the iterative reconstruction, among which the ordered-subset (OS) scheme is very attractive. As a result, the SART algorithm can be accelerated by the OS scheme. This combination is called OS-SART [23, 24]. To formulate an OS version of the SART technique, we assume that the index set *B* = {1,…, *M*} can be partitioned into *T* nonempty disjoint subsets *B*_*t*_ = {*i*_1_^*t*^,…, *i*_*M*(*t*)_^*t*^} such that

(1) $$\begin{array}{c} B=\left\{1,\ldots ,M\right\}=\overset{}{\underset{1\leq t\leq T}{⋃}}B_{t}. \end{array}$$

Then, a possible version of the OS-SART formulation can be expressed as

(2) $$\begin{array}{c} {f_{j}}^{\left(n+1\right)}={f_{j}}^{\left(n\right)}+\sum_{i\in B_{\left[n\right]}}^{}\frac{a_{ij}}{a_{+j}}\frac{b_{i}-A^{i}f^{\left(n\right)}}{a_{i+}}, \end{array}$$

where *a*_*i*+_ ≡ ∑_*j*=1_^*N*^ *a*_*ij*_ ≠ 0,  *a*_+*j*_ ≡ ∑_*i*=1_^*M*^ *a*_*ij*_ ≠ 0. 

The above OS-SART reconstruction method can be empowered by the CS technique to improve the image quality under less favorable measurement conditions. As mentioned earlier, the discrete gradient transform (DGT) is a valid sparse transform for dental images. Hence, a dentin image can be reconstructed from truncated projections data via the *ℓ*_1_-norm minimization of the DGT, which is the TV minimization [19, 28]. Mathematically, it can be modeled as

(3) $$\begin{array}{c} \underset{f}{\min}{||\nabla f||}_{1},\;\text{subject}\;\;\text{to}\;Af=b,\;f>0, \end{array}$$

where ||∇*f*||_1_ denotes TV of *f*, and

(4) $$\begin{array}{c} {||\nabla f||}_{1}=\sum_{i,j}d_{i,j},\;d_{i,j}=\sqrt{{\left(f_{i,j}-f_{i+1,j}\right)}^{2}+{\left(f_{i,j}-f_{i,j+1}\right)}^{2}}, \end{array}$$

where  *f*_*i*,*j*_  is a pixel value of the discrete 2D image and *d*_*i*,*j*_  is a discrete gradient. 

 Equation (3) can be implemented in two loops. The outer loop implements OS-SART to reduce data discrepancy, and the inner loop minimizes the image TV. In the inner loop, we use the gradient descent method:

(5) $$\begin{array}{c} f^{\left(m+1\right)}=f^{\left(m\right)}-\lambda \omega \upsilon , \end{array}$$

where  *λ* is a gradient descent control coefficient, *υ* = (∂||∇*f*||_1_/∂*f*_*i*,*j*_)∣_*f*_*i*,*j*_=*f*_*i*,*j*_[*n*,*m*]_ is a gradient direction with *f*_*i*,*j*_ = *f*_*i*,*j*_[*n*, *m*], *ω* = max⁡(|*f* ^(*m*)^|)/max⁡(|*υ*|) is a scaling coefficient of the gradient descent and n and m are the outer and inner loop iteration indices, respectively. 

The whole iteration process can be summarized in the following steps.

**Step 1**

Input measured data *b* and an initial image  *f* = 0.

**Step 2**

Update the current image using OS-SART by (2).

**Step 3**

Minimize the TV of the current image using the gradient descent method by (5).

**Step 4**

Go to Step 2 until a stopping criterion is met.

In our implementation, the gradient descent control coefficient was  *λ* = 0.2, the TV iteration number was  *m* = 30, and the OS-SART iteration number was  *n* = 20.

## 3. Results and Analysis

### 3.1. Numerical Simulation

**Figure 3:**
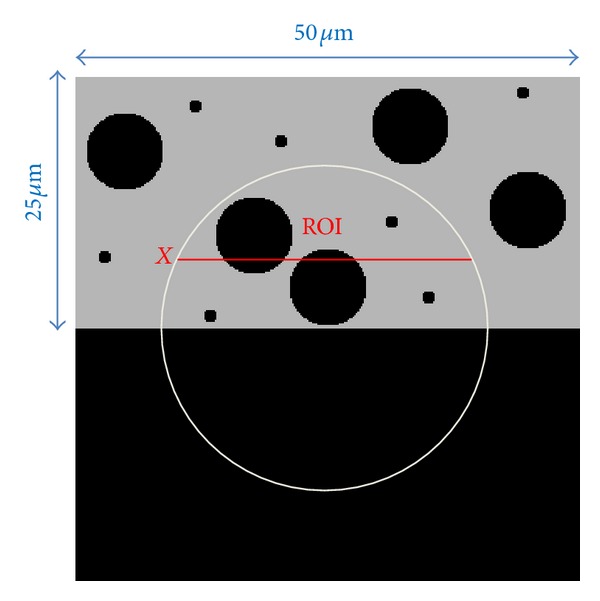
Dentin phantom. The circular region indicates a ROI, with the line labeled “X” for subsequent profiling.

To evaluate the performance of interior tomography for studying the dentin specimen, we designed a dentin phantom as shown in Figure 3. This phantom has two distinct sizes of pores representing tubules and canaliculi. The dentin phantom was made 25 *μ*m × 50 *μ*m in size and discretized into a 150 × 300 matrix (the pixel size: 0.17 *μ*m × 0.17 *μ*m). The tubules and canaliculi outside a prespecified ROI represent structures that might affect the interior reconstruction. Because the dentin composition is similar to cortical bone, we used cortical bone to mimic dentin attenuation characteristics in the simulation. The linear attenuation coefficient of cortical bone was estimated as 117 cm^−1^ for an X-ray energy 8 keV according to the X-ray Attenuation Databases reported by the National Institute of Standards and Technology (NIST). The scanning range was −70° to +70° (0° is for the normal to the plate-like specimen) with either a 0.25° or 1° angular increment and captured two groups of truncated projection data (a total of 561 or 141 projections, resp.). We then used FBP and CS-based interior tomography methods, respectively, to reconstruct the ROI from the two datasets for comparison.

**Figure 4:**
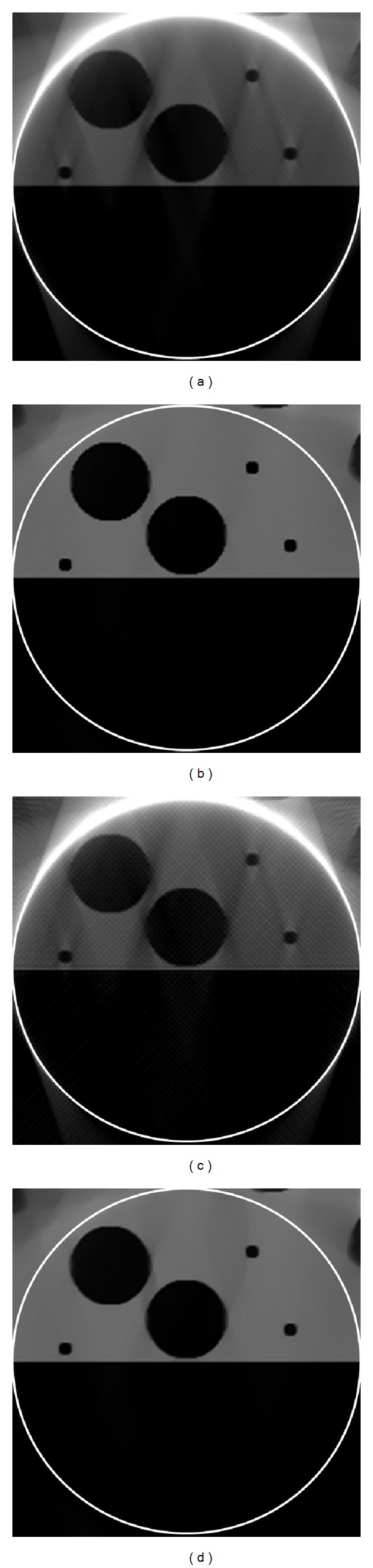
Reconstruction results. (a) The reconstructed ROI using FBP from 561 projections, (b) the reconstructed ROI using interior tomography from 561 projections, (c) the reconstructed ROI using FBP from 141 projections, and (d) the reconstructed ROI using interior tomography from 141 projections. The display window is [0, 585] cm^−1^.

**Figure 5:**
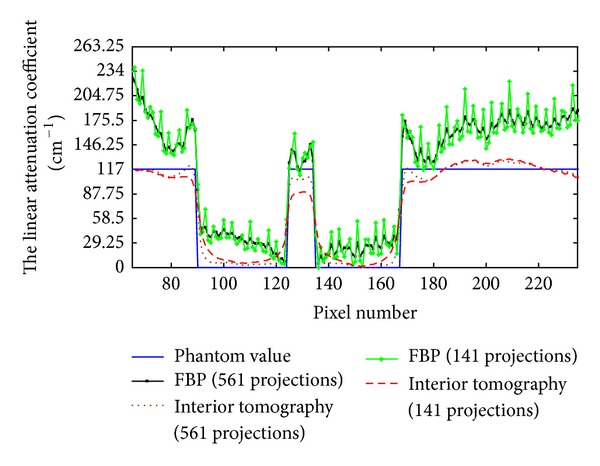
Profiles corresponding to the line “X” in Figure 3.

The reconstructed results are in Figure 4. It can be seen in Figure 4 that there were some streak and shadow artifacts in the reconstructed images using FBP from truncated projections, and the interior tomography method could suppress these artifacts effectively.  Figure 5 shows the profiles along the line “X” in Figure 4.

**Figure 6:**
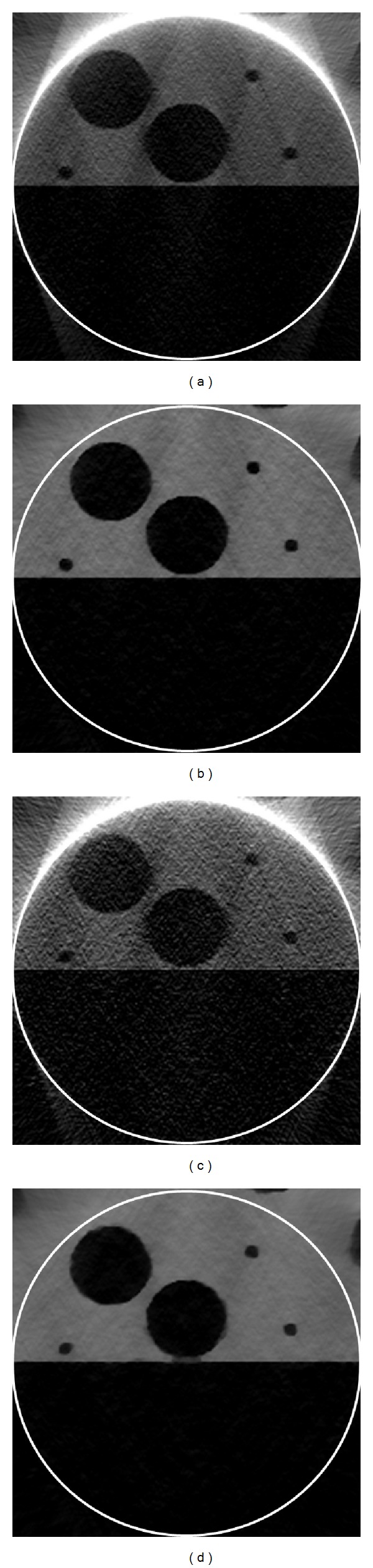
Reconstruction results from the data with 1% Gaussian noise. (a) The reconstructed ROI using FBP from 561 projections, (b) the reconstructed ROI using interior tomography from 561 projections, (c) the reconstructed ROI using FBP from 141 projections, and (d) the reconstructed ROI using interior tomography from 141 projections. The display window is [0, 585] cm^−1^.

**Figure 7:**
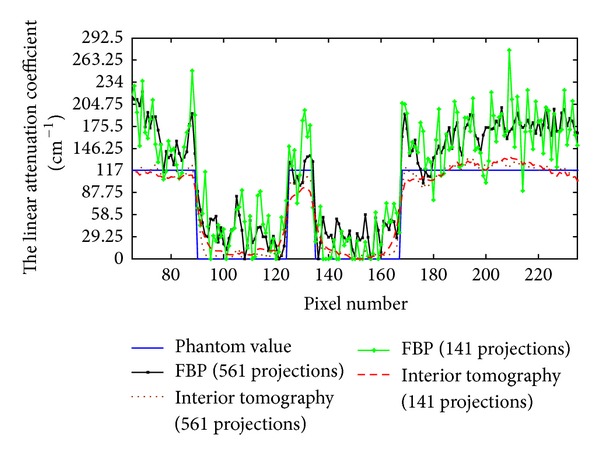
Profiles corresponding to the line “X” in Figure 3.

To test the stability of interior tomography against data noise, we repeated the reconstructions from projections contaminated with 1% Gaussian noise level. The reconstructed results from the data with 1% Gaussian noise are in Figure 6. It can be seen that there were strong noises in the images reconstructed using FBP from noise projections, and interior tomography could suppress these noises well.  Figure 7 shows representative profiles corresponding to Figure 6, along the line “X” in Figure 3.

Then, we used the root mean square error (RMSE) to quantify the reconstructed results, which is expressed as

(6) $$\begin{array}{c} \text{RMSE}=\sqrt{\frac{\sum_{i,j\in \text{ROI}}^{}{\left(\mu_{i,j}-{\hat{\mu }}_{i,j}\right)}^{2}}{N_{\text{ROI}}}}, \end{array}$$

where ${\hat{\mu }}_{i,j}$  is the reconstructed pixel value, *μ*_*i*,*j*_  is the true value of the phantom, and  *N*_ROI_ is the number of the pixels in the ROI. The RMSE values are in Table 1.

### 3.2. Experimental Study

**Figure 8:**
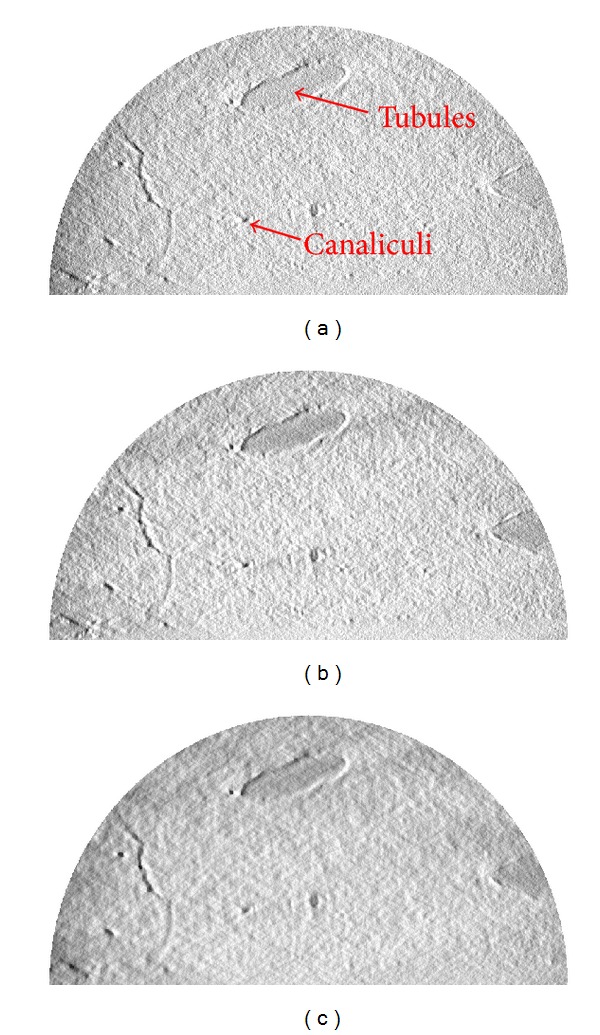
ROI reconstruction of the dentin specimen. (a) The reconstruction using FBP from 561 projections, (b) The reconstruction using interior tomography from 561 projections, and (c) the reconstruction of interior tomography from 141 projections. The display window is consistent.

Supported by our encouraging numerical results, we applied the interior tomography method to study the dentin specimen.  Figure 8 compares the reconstructions of the dentin specimen with FBP and interior tomography. The interior tomographic reconstructions were performed from 561 and 141 projections, respectively. Similar to simulation analysis, there were some noise and artifacts in the reconstructed results from actual data. In theory, the pixel values in canaliculi and tubules regions should be similar and smaller than that of dentin. From the FBP reconstruction (Figure 8(a)), the gray values of the tubule region can be found, which may be imperfect due to data noise (higher brightness). Quantitatively, the slices reconstructed using interior tomography produced less noises and artifacts than the FBP image, and interior tomography has better stability than FBP.

**Figure 9:**
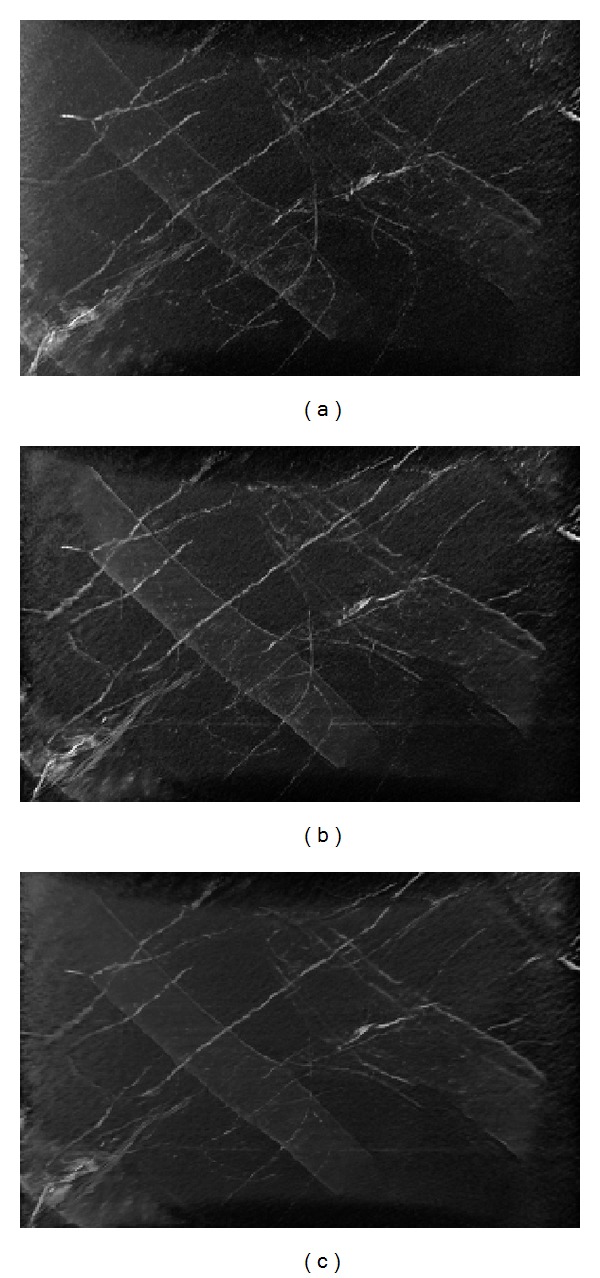
Reconstructed dentin structures. (a) The volume rendering based on the FBP reconstruction from 561 projections, (b) the volume rendering based on the interior tomographic reconstruction from 561 projections, and (c) the volume rendering based on the interior tomographic reconstruction from 141 projections. The 3D visualization display window is consistent.

To analyze the internal microstructures, two volumes of 600 high-resolution dentin slices were reconstructed using interior tomography from 561 projections and 141 projections, respectively. As a benchmark, a volume of the same 600 high-resolution dentin slices was also reconstructed using FBP from the 561 projections. All of these image volumes were rendered, as shown in Figure 9.  Figure 9(a) shows the 3D result using FBP from truncated projections. In the interior tomographic reconstructions from truncated projections, noises and artifacts were significantly suppressed, producing 3D visualization with a better signal-to-noise ratio, even with only 141 projections, as shown in Figures 9(b) and 9(c).

## 4. Discussions and Conclusion

For high-resolution image reconstructions, the FBP algorithm is very efficient and accurate. With truncated datasets, however, the FBP method is subject to more noises and artifacts than those reconstructed by the iterative approach. In the piecewise-constant-model-based interior tomography framework, we have employed several techniques to increase convergence rate while improving image quality. First, an OS version of the Landweber scheme has been used. Second, the code has been optimized, combining the merits of C++ and multicore techniques. Third, a high-performance computer has been utilized to run our code program. Particularly, we have simultaneously reconstructed 8 slices using 8 central processing units (CPU). 

The CS theory indicates that an image can be often accurately reconstructed from a rather limited amount of data when it can be sparsely represented in an appropriate domain. The internal feature of the dentin specimen is complex, and porosity is characteristic. We consider a dentin object approximately piecewise constant. Then, a dentin image is sparsified by a discrete gradient transform. Because the dentin projections are intrinsically truncated, it is inevitable that there are some artifacts in the image reconstructed using the FBP method. On the other hand, interior tomography is shown to be promising in meeting the challenge. In particular, the ability to generate a volume rendering with a high signal-to-noise ratio from a very limited number of truncated projections is quite feasible using interior tomography.

For real data study, our purpose is to reconstruct a high-quality dentin image. For 2D image reconstruction, there were some shadows (lower brightness) in the canaliculi and tubules regions reconstructed by the CS-based interior tomography method, which could reflect the attenuation characteristics of dentin interior structure. However, for 3D image reconstruction, the CS-based interior tomography method could suppress image artifacts and noises for the reconstructed images from truncated projections. Moreover, the CS-based interior tomography minimizes the TV of a reconstructed image by the steepest gradient descent method to generate a better looking 3D perspective view, which might oversmooth fine details if the number of views is too small. In the future studies we will analyze more dentin specimens to evaluate the performance of interior tomography and use the dictionary learning technique to capture more information. 

In conclusion, we have developed a piecewise-constant-model-based interior tomography method to deal with truncated projections collected over a limited angular range, and investigated the feasibility and potential of the interior tomographic application in dentin characterization. It has been demonstrated that the CS-based interior tomography method is advantageous for the dentin reconstruction from incomplete nano-CT data. Further improvements are underway to facilitate dental research.

## Figures and Tables

**Table 1:** RMSE values for the reconstructed ROI images.

Reconstruction protocol	Noise-free data	Data with 1% noise
FBP (561 projections)	117.00	122.85
FBP (141 projections)	140.40	152.10
Interior tomography (561 projections)	9.65	10.59
Interior tomography (141 projections)	11.81	12.75
